# Parathyroid-Specific Deletion of *Klotho* Unravels a Novel Calcineurin-Dependent FGF23 Signaling Pathway That Regulates PTH Secretion

**DOI:** 10.1371/journal.pgen.1003975

**Published:** 2013-12-12

**Authors:** Hannes Olauson, Karolina Lindberg, Risul Amin, Tadatoshi Sato, Ting Jia, Regina Goetz, Moosa Mohammadi, Göran Andersson, Beate Lanske, Tobias E. Larsson

**Affiliations:** 1 Division of Renal Medicine, Department of Clinical Science, Intervention and Technology, Karolinska Institutet, Stockholm, Sweden; 2 Department of Oral Medicine, Infection, and Immunity, Harvard School of Dental Medicine, Boston, Massachusetts, United States of America; 3 Department of Biochemistry and Molecular Pharmacology, New York University School of Medicine, New York, New York, United States of America; 4 Division of Pathology, Department of Laboratory Medicine, Karolinska Institutet and Karolinska University Hospital Huddinge, Stockholm, Sweden; 5 Department of Nephrology, Karolinska University Hospital, Stockholm, Sweden; Yale University, United States of America

## Abstract

Klotho acts as a co-receptor for and dictates tissue specificity of circulating FGF23. FGF23 inhibits PTH secretion, and reduced Klotho abundance is considered a pathogenic factor in renal secondary hyperparathyroidism. To dissect the role of parathyroid gland resident Klotho in health and disease, we generated mice with a parathyroid-specific *Klotho* deletion (*PTH-KL^−/−^*). *PTH-KL^−/−^* mice had a normal gross phenotype and survival; normal serum PTH and calcium; unaltered expression of the *PTH* gene in parathyroid tissue; and preserved PTH response and sensitivity to acute changes in serum calcium. Their PTH response to intravenous FGF23 delivery or renal failure did not differ compared to their wild-type littermates despite disrupted FGF23-induced activation of the MAPK/ERK pathway. Importantly, calcineurin-NFAT signaling, defined by increased MCIP1 level and nuclear localization of NFATC2, was constitutively activated in *PTH-KL^−/−^* mice. Treatment with the calcineurin-inhibitor cyclosporine A abolished FGF23-mediated PTH suppression in *PTH-KL^−/−^* mice whereas wild-type mice remained responsive. Similar results were observed in thyro-parathyroid explants *ex vivo*. Collectively, we present genetic and functional evidence for a novel, Klotho-independent, calcineurin-mediated FGF23 signaling pathway in parathyroid glands that mediates suppression of PTH. The presence of Klotho-independent FGF23 effects in a Klotho-expressing target organ represents a paradigm shift in the conceptualization of FGF23 endocrine action.

## Author Summary

Inorganic calcium is a critical element for a diverse range of cellular processes ranging from cell signaling to energy metabolism, and its extracellular concentration is controlled by parathyroid hormone (PTH). Klotho is expressed in parathyroid chief cells and reported to facilitate PTH secretion during hypocalcemia and mediate FGF23 suppression of PTH synthesis and secretion. To dissect the role of parathyroid Klotho in health and disease, we generated parathyroid-specific *Klotho* knockout mice. The mutant mice had normal serum levels of PTH and calcium. Further, their parathyroid sensitivity to acute fluctuations in serum calcium and response to FGF23 treatment were preserved, and mutant mice developed secondary hyperparathyroidism of similar magnitude as wild-type mice when challenged with renal failure. A previously unknown parathyroid FGF23 signaling pathway involving calcineurin was constitutively activated in the mutant mice, and blocking this pathway abolished FGF23-induced suppression of PTH secretion. Our data challenges the concepts of Klotho as a mandatory factor for the acute hypocalcemic PTH response and decreased Klotho abundance as a pathogenic factor in secondary hyperparathyroidism. Finally, the presence of Klotho-independent FGF23 effects in a Klotho-expressing target organ represents a paradigm shift in the conceptualization of FGF23 endocrine action.

## Introduction

Calcium plays a pivotal role in many biological processes, such as intra-cellular signaling, cell membrane depolarization and excitation, energy metabolism and skeletal mineralization. Accordingly, a fine-tuned regulation of serum calcium level is a prerequisite for normal cellular and organ function in most organisms. Parathyroid hormone (PTH) is the principal hormonal regulator of circulating calcium as it rapidly increases its renal tubular reabsorption and mobilization from bone deposits in response to a decrease in serum calcium [1]. In turn, free calcium ions can efficiently inhibit PTH secretion as part of an endocrine feedback loop mediated by the calcium-sensing receptor (CaSR) located on parathyroid chief cells [2].

Type I membrane-bound alpha-Klotho (Klotho) defines tissue specificity for the phosphaturic hormone fibroblast growth factor-23 (FGF23) by acting as a permissive co-receptor [3]. Klotho is predominantly expressed in organs requiring abundant calcium transport such as kidneys, parathyroid glands and choroid plexus [4]. In the parathyroids, FGF23 binds to binary complexes of an FGF receptor (FGFR) and Klotho to suppress PTH secretion [5], [6]. Klotho activity on the other hand has been implicated as fundamental for the stimulation of PTH secretion during hypocalcemic conditions [7], although the underlying mechanism has been challenged [8].

Secondary hyperparathyroidism (sHPT) is a common manifestation in chronic kidney disease (CKD) despite markedly increased serum FGF23 concentrations. This presumably reflects parathyroid resistance to FGF23 action, which was also supported by lack of response to FGF23 injections in a rat model of CKD [9], [10]. The proposed mechanism underlying such FGF23 resistance is decreased abundance of parathyroid Klotho and FGFRs [11], [12].

To dissect the role of parathyroid gland resident Klotho in physiology and in pathophysiological states such as CKD, we generated a novel mouse strain harboring a parathyroid-specific deletion of the *Klotho* gene. The present study sheds new light on the function of parathyroid Klotho and identifies a novel, Klotho-independent signaling pathway of FGF23 that is involved in the regulation of PTH secretion.

## Results

### Generation of *PTH-KL^−/−^* mice

**Figure 1:**
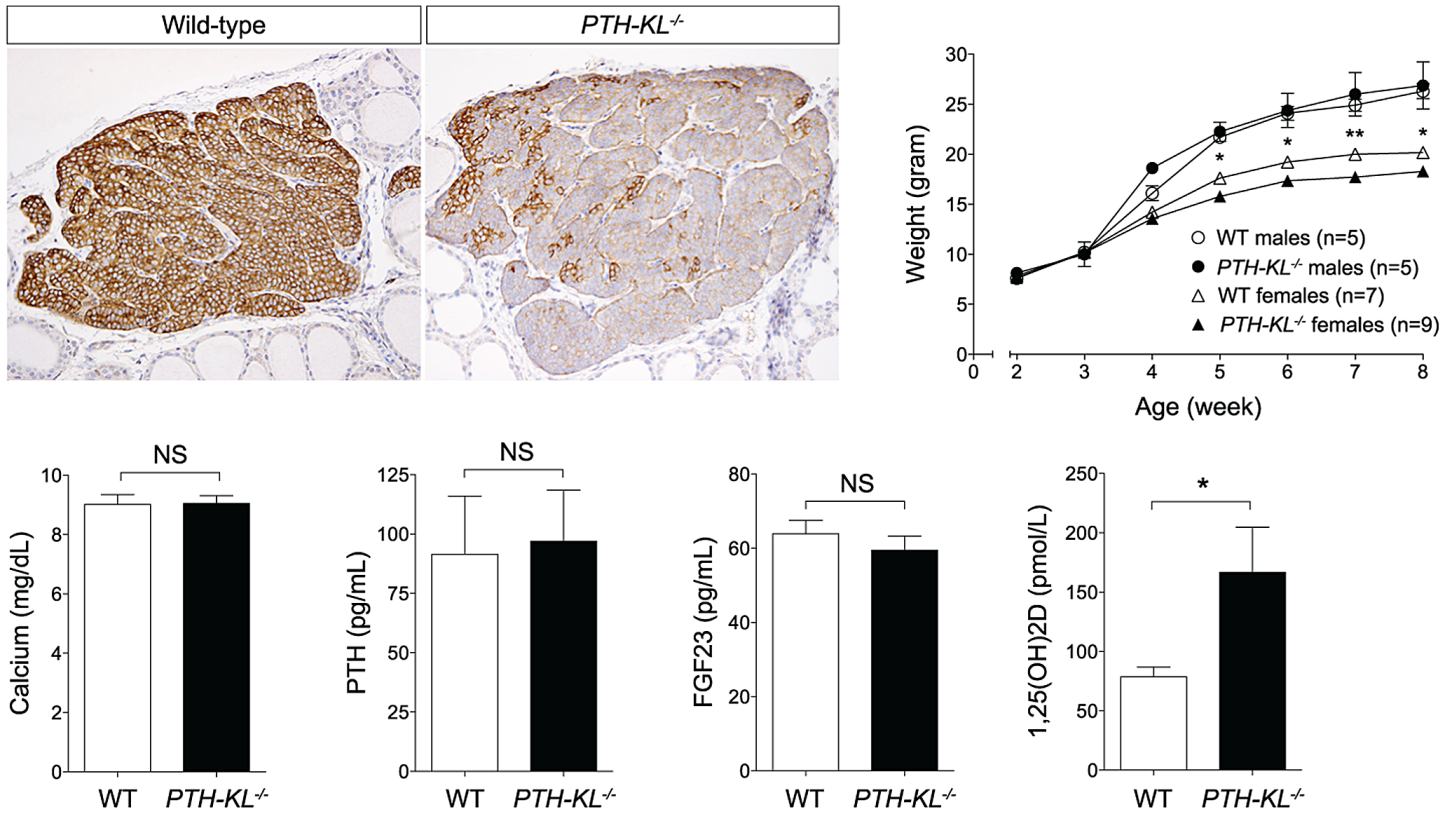
Tissue-specific deletion of the *Klotho* gene. *Upper left panel*. Immunohistochemical staining confirmed successful deletion of Klotho in parathyroid glands of *PTH-KL^−/−^* mice. 20× magnification. *Upper right panel*. The gross appearance of *PTH-KL^−/−^* mice was normal although females had lower body weight and shorter crown-rump length compared to wild-type littermates. No differences were seen for male *PTH-KL^−/−^* mice. *p<0.05 **<0.01. *Lower panel:* Serum levels of 1,25(OH)_2_D were doubled in *PTH-KL^−/−^* mice compared to their wild-type littermates (p<0.05), while PTH, calcium and FGF23 remained normal.

Mice with a parathyroid specific deletion of Klotho (*PTH-KL^−/−^*) were generated using Cre-LoxP recombination (Figure S1). Floxed Klotho mice were crossed with mice expressing Cre recombinase driven by the human PTH promoter, which was previously shown to have Cre activity exclusively in the parathyroid glands [13]. Successful deletion of parathyroid Klotho protein was confirmed with immunohistochemical staining of thyro-parathyroid tissue (Figure 1). Overall efficiency of deletion varied, and was up to >90% in investigated samples. Subanalyses of mice with the most efficient deletion showed similar results to the full analyses.

### Gross phenotype and survival of *PTH-KL^−/−^* mice

Adult *PTH-KL^−/−^* mice were viable, fertile and did not display any gross physical or behavioral abnormalities. Survival was similar to wild-type littermates during the study with no mortality up to 6 months of age. Female *PTH-KL^−/−^* mice had reduced body weight and crown-rump length compared to wild-type littermates (p<0.05; Figure 1), whereas no such differences were found in male *PTH-KL^−/−^* mice.

### Serum and urine biochemistries of *PTH-KL^−/−^* mice

Serum biochemistries in 8-week-old mice are shown in Figure 1. Interestingly, the serum levels of 1,25-dihydroxy vitamin D_3_ (1,25(OH)_2_D) were doubled in *PTH-KL^−/−^* mice compared to their wild-type littermates (p<0.05), while PTH, calcium and FGF23 remained normal. Serum phosphorous (wild-type vs *PTH-KL^−/−^*; 9.54±0.38 vs 9.39±0.32, p>0.05), creatinine (0.31±0.03 vs 0.26±0.02, p>0.05), urinary concentrations of calcium/creatinine (0.53±0.19 vs 0.53±0.19, p>0.05) and phosphorous/creatinine (19.1±3.4 vs 19.4±3.0, p>0.05) were also unaltered in *PTH-KL^−/−^* mice. To exclude the possibility of early onset changes in mineral metabolism, we analyzed serum from 3-week-old animals. No differences were seen for serum calcium (wild-type vs *PTH-KL^−/−^*; 10.00±0.22 vs 9.86±0.20, p>0.05) or PTH (118.0±13.4 vs 133.0±21.8, p>0.05).

### Bone and renal phenotype of *PTH-KL^−/−^* mice

We investigated potential secondary effects that the parathyroid-specific deletion of the *Klotho* gene might have on the main target organs of PTH signaling, namely bone and kidney. There were no significant histological changes in bone from 6-week old *PTH-KL^−/−^* mice and bone mineral density was unaltered compared to wild-type mice (Figure S2). Renal histology was also normal and no transcriptional changes were found for *Klotho*, *VDR*, *Cyp27b1*, *Cyp24a1*, *Npt2a*, *TRPV5* or *CaSR* in kidneys from *PTH-KL^−/−^* mice compared to wild-type mice (Table S1).

### Parathyroid histology and protein expression in *PTH-KL^−/−^* mice

Parathyroid size, histology and proliferation index, defined as Ki67 positive cells/total number of cells, was not affected by the *Klotho* gene deletion (wild-type vs *PTH-KL^−/−^*, 2.9% vs. 2.8%, p>0.05) (Figure S3A). Immunofluorescence staining showed no significant changes in protein expression for PTH, CaSR, VDR (Figure S3B), FGFR1 or Cyp27b1 (data not shown) suggesting that Klotho does not regulate the expression of these proteins in an autocrine or paracrine fashion under physiological conditions.

### Expression of genes critical for parathyroid function in *PTH-KL^−/−^* mice

**Figure 2:**
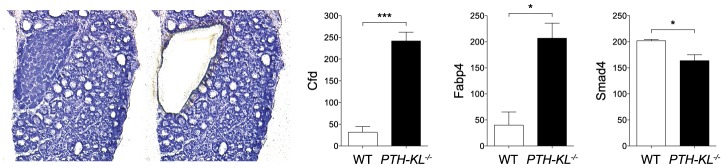
Parathyroid transcriptional data. Transcriptional analysis of laser microdissected parathyroid tissue revealed differential expression of *Cfd*, *Smad4* and *Fabp4* in *PTH-KL^−/−^* mice (n = 3 for each genotype). *p<0.05 ***p<0.001.

The expression of approximately 90 genes critical for parathyroid function was examined using a nanostring array. The data are compiled in Table S2. In addition to *Klotho*, changes in expression level were observed for genes important for transcriptional and metabolic control, such as *Cfd* (Entrez Gene: 11537), *Fabp4* (11770) and *Smad4* (17128)(Figure 2). *Gli3* (14634), *Pin1* (23988), *sFRP3* (20379) and *FGF20* (80857) were also expressed at different levels in the parathyroids of *PTH-KL^−/−^* mice compared to wild-type mice, although at a low absolute level. Notably, the expression of some of the genes that play a key role in the function of the parathyroid gland, such as *PTH*, *Gata3*, *CaSR* and *VDR*, or in FGF23 physiology, such as *FGFR1* and the vitamin D regulatory enzymes *Cyp27b1* and *Cyp24a1*, were not significantly affected by the knockdown of parathyroid *Klotho* gene expression.

### Parathyroid sensitivity and response to rapid changes in serum calcium in *PTH-KL^−/−^* mice

The serum calcium level of *PTH-KL^−/−^* and wild-type mice was either rapidly decreased or increased by an intraperitoneal injection of EGTA or calcium-gluconate, respectively [14]. The parathyroid response to alterations in serum calcium, as measured by serum PTH level, did not differ between *PTH-KL^−/−^* mice and wild-type mice (wild-type vs *PTH-KL^−/−^*, R^2^ = 0.77, p<0.0001 vs R^2^ = 0.74, p<0.0001)(Figure S4), supporting that parathyroid Klotho is not essential for parathyroid sensitivity to acute changes in serum calcium, and its response to them with altered PTH secretion.

### Parathyroid response to induction of renal failure in *PTH-KL^−/−^* mice

**Figure 3:**
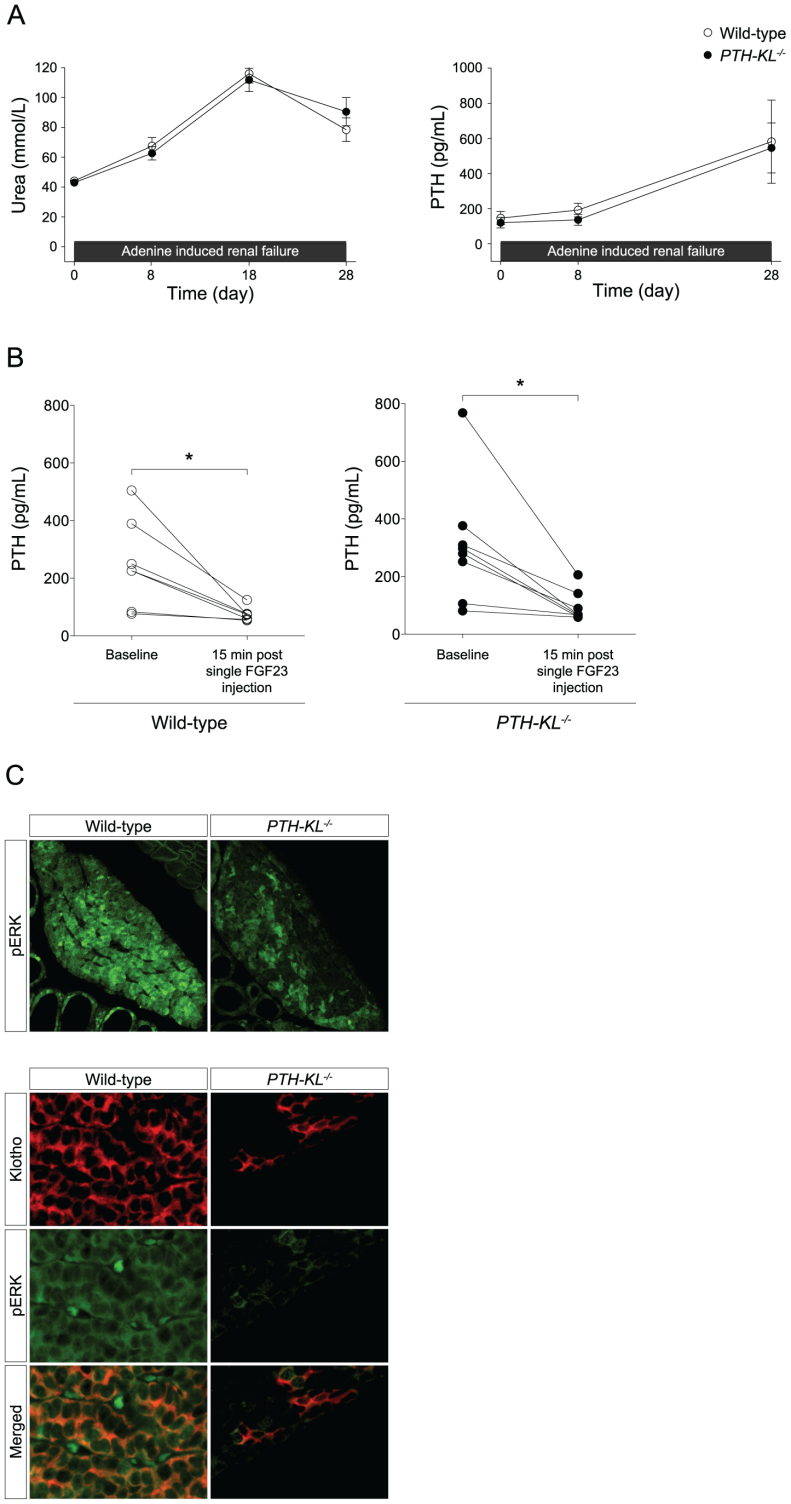
Responsiveness to renal failure and a single FGF23 injection. A) Mice were challenged with renal failure during 4 weeks by dietary adenine delivery. *PTH-KL^−/−^* (n = 8) and wild-type (n = 6) mice developed uremia and secondary hyperparathyroidism of similar magnitude. B) Mice were injected intravenously with recombinant FGF23 protein at the dose 0.15 mg/kg and blood samples from the tail vein were drawn after 15 minutes. *PTH-KL^−/−^* mice had a similar rapid reduction in PTH followed by a single FGF23 injection indicating a preserved FGF23 responsiveness in *PTH-KL^−/−^* mice despite ablated Klotho expression. Figure shows pooled data from two independent experiments (n = 9 for *PTH-KL^−/−^*; n = 7 for wild-type mice). *p<0.05 C) The impact of Klotho-dependent FGF23 signaling was investigated in parathyroid glands with dual immunofluorescence staining of Klotho and phosphorylated ERK1/2. Importantly, phosphorylated ERK1/2 was strongly induced 15 minutes after a single intravenous FGF23 injection in wild-type mice. In contrast, *PTH-KL^−/−^* mice failed to phosphorylate ERK1/2 specifically in cells lacking Klotho, supporting a disruption of functional Klotho-mediated FGF23 signaling in *PTH-KL^−/−^* mice (10× and 40× magnification).

Renal insufficiency is associated with the development of sHPT. To test whether parathyroid function is different in *PTH-KL^−/−^* mice under conditions of renal insufficiency, we induced renal failure using an adenine-based protocol [15]. Four weeks after induction of renal insufficiency, *PTH-KL^−/−^* mice exhibited sHPT just like their wild-type littermates did. Its severity as well as changes in other markers of mineral metabolism was similar among *PTH-KL^−/−^* and wild-type mice (Figure 3A and Table S3). Notably, serum FGF23 levels were increased by approximately 50-fold in both *PTH-KL^−/−^* and wild-type mice with renal failure compared to mice with preserved renal function.

### Parathyroid response to FGF23 in *PTH-KL^−/−^* mice

Based on our findings that the parathyroid glands of *PTH-KL^−/−^* mice retained calcium sensitivity and that sHPT developed similarly as in wild-type mice upon induction of renal insufficiency, we reasoned that the parathyroid response to FGF23 might also be preserved in *PTH-KL^−/−^* mice. To test this, we injected mice intravenously with a single dose of recombinant FGF23 and measured serum concentrations of PTH before and 15 min after the injection. As shown in Figure 3B, FGF23 caused a decrease in serum levels of PTH in *PTH-KL^−/−^* mice of similar magnitude as in wild-type mice.

### FGF23 signaling pathway in the parathyroid glands of *PTH-KL^−/−^* mice

We next explored which signaling pathway might mediate the inhibition by FGF23 on PTH secretion in *PTH-KL^−/−^* mice. Activation of the MAPK cascade was first examined using immunofluorescence staining. As shown in Figure 3C, there was significantly less immunostaining for phosphorylated ERK1/2 in parathyroid tissue from *PTH-KL^−/−^* mice, and a complete co-localization with residual Klotho protein. These data indicate that activation of the MAP kinase pathway by FGF23 is markedly suppressed or absent in Klotho-deficient parathyroids.

**Figure 4:**
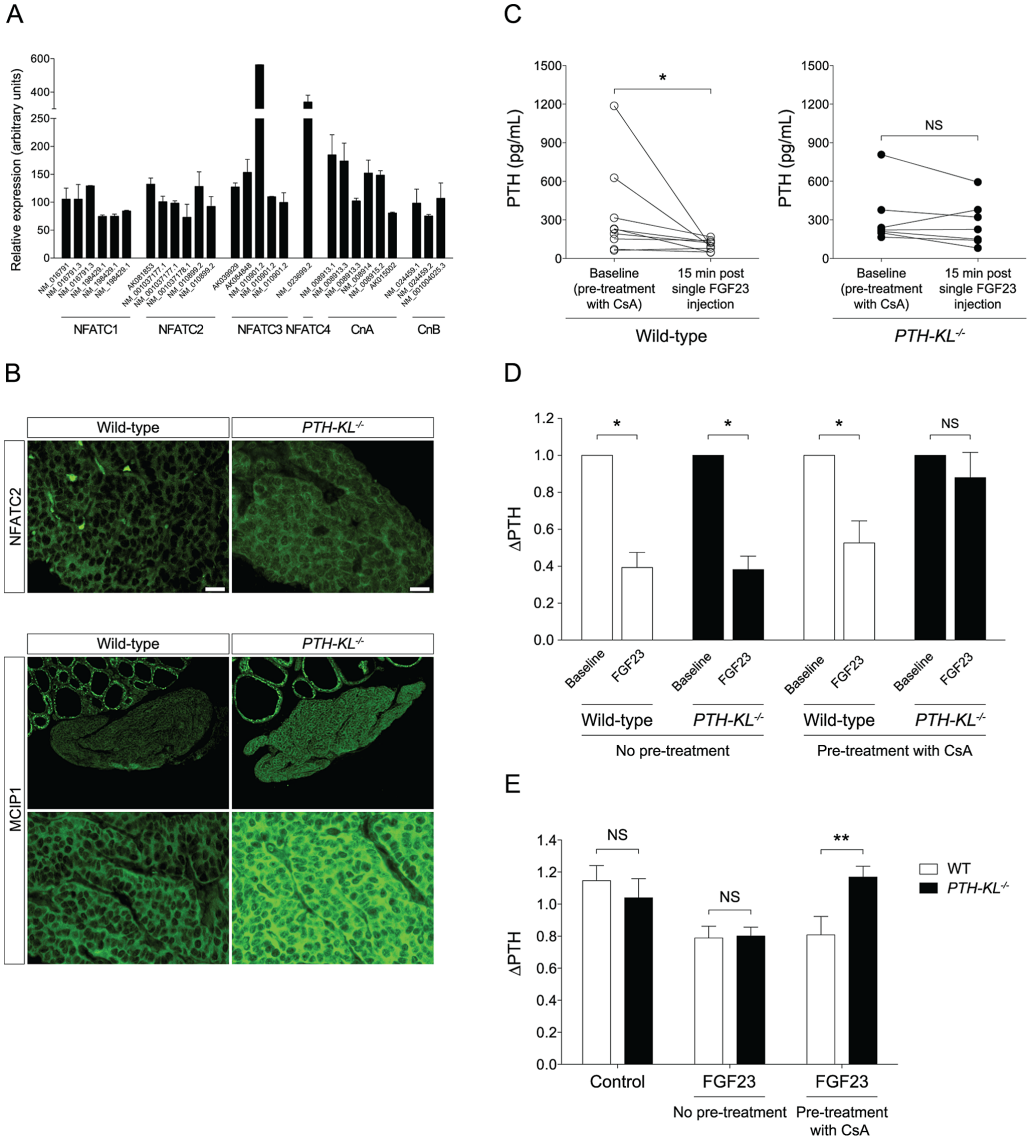
Presence of the calcineurin-NFAT pathway and the impact of cyclosporin A on FGF23 treatment. A) Transcript analysis of laser capture microdissected parathyroid glands from 3- week-old wild-type mice revealed presence of NFATC1-4 and the subunits for calcineurin A and B (CnA and CnB). Each bar represents one hybridization probe. Data is presented as mean (± SEM). B) Immunostaining for NFATC2 showed a partial nuclear translocation in *PTH-KL^−/−^* mice, and cytoplasmatic localization in wild-type mice after treatment with FGF23, indicating activation of the calcineurin-NFAT pathway in parathyroids of *PTH-KL^−/−^* mice. In addition, MCIP1, a facilitator of calcineurin signaling, was markedly higher in *PTH-KL^−/−^* compared to wild-type mice after treatment with FGF23 (10× and 40× magnification). C) Pretreatment of mice with the calcineurin inhibitor CsA followed by a single intravenous FGF23 injection. Wild-type mice had a preserved responsiveness to FGF23 as evidenced by a significant reduction in serum PTH 15 minutes after FGF23 injection (p<0.05) whereas FGF23-mediated inhibition of serum PTH was blunted in *PTH-KL^−/−^* mice. Figure depicts pooled data from two independent experiments, using 60 mg/kg and 150 mg/kg CsA respectively, with similar results. Total n = 10 for wild-type and 7 for *PTH-KL^−/−^* mice. *p<0.05. D) Relative change in PTH level in wild-type and *PTH-KL^−/−^* mice 15 min after a single FGF23 injection, with and without pretreatment of CsA. E) Thyro-parathyroid explants were cultured in serum-free medium and exposed to FGF23 for 2 h with or without pre-treatment by CsA. Wild-type and *PTH-KL^−/−^* mice responded with a similar decrease in PTH when exposed to FGF23. In contrast, in explants pre-treated with CsA (0.83 µM) for 2 h the response to FGF23 treatment was unaltered in wild-type mice but completely blunted in *PTH-KL^−/−^* mice. N = 5–10 for all groups. **p<0.01.

We next tested whether the calcineurin-nuclear factor of activated T cells (NFAT) pathway, another proposed downstream signaling pathway of FGFR activation [16], [17], [18], was activated in the parathyroids of *PTH-KL^−/−^* mice in response to FGF23. To this end, we analyzed gene expression of calcineurin and NFAT in parathyroid tissue from wild-type mice. Indeed, all subunits of calcineurin and all four calcium-regulated members of the NFAT family (NFATC1–NFATC4) were expressed in the parathyroid of wild-type mice (Figure 4A). We then examined by immunofluorescence microscopy the subcellular distribution of the NFAT proteins in parathyroid tissue from *PTH-KL^−/−^* mice that had been treated with FGF23. While parathyroid tissue from FGF23 treated wild-type mice showed cytoplasmic immunostaining for NFATC2, the parathyroid tissue of FGF23 treated *PTH-KL^−/−^* mice also showed nuclear localization of NFATC2 (Figure 4B). No clear difference was seen for the other NFATs. In addition, immunostaining analysis for the modulatory calcineurin interacting protein 1 (MCIP1), a facilitator of calcineurin activity, revealed that MCIP1 expression was markedly upregulated in Klotho-deficient parathyroid tissue compared to wild-type tissue (Figure 4B) [19]. Together, the data suggest that the calcineurin-NFAT pathway is activated in the parathyroid of *PTH-KL^−/−^* mice, and responds to treatment with FGF23.

To provide *in vivo* evidence for the role of the calcineurin-NFAT pathway in mediating the suppression by FGF23 of PTH secretion, *PTH-KL^−/−^* and wild-type mice were pre-treated with cyclosporine A (CsA), a calcineurin-inhibitor, prior to injection of FGF23. As shown in Figures 4C and D, the CsA pre-treatment nearly abolished the effect of FGF23 on PTH secretion in *PTH-KL^−/−^* mice, whereas it did not impact this FGF23 action in wild-type mice.

To investigate whether the parathyroid calcineurin-NFAT pathway acts independent of serum factors such as soluble Klotho, thyro-parathyroid explants from wild-type and *PTH-KL^−/−^* mice were treated with FGF23 with or without pre-treatment of CsA [20]. In explants without pretreatment of CsA, 2 h treatment with FGF23 (10 ng/mL) decreased PTH secretion similarly in wild-type and *PTH-KL^−/−^* mice (Figure 4E). Conversely, in explants pre-treated with CsA (0.83 µM) for 2 h the response to FGF23 treatment was unaltered in wild-type mice but completely blunted in *PTH-KL^−/−^* mice (ΔPTH relative to baseline; 0.81 vs 1.17, p<0.01).

## Discussion

Regulation of PTH secretion is a fundamental process in calcium homeostasis, yet our current models are constantly revised due to the complex and multi-dimensional control of PTH. Recent data suggest that, in addition to calcium-sensing receptor and vitamin D receptor activation, the FGF23-Klotho endocrine axis negatively regulates PTH secretion. However, previous discordant results of FGF23-Klotho function in physiology and in pathophysiological states such as renal sHPT prompted us to further investigate the concerted parathyroid action of FGF23-Klotho *in vivo*. We addressed this issue by generating a novel mouse model harboring a parathyroid-specific deletion of *Klotho* and our principal findings are that i) absence of parathyroid Klotho does not alter the acute parathyroid response of PTH secretion and calcium sensitivity as previously suggested; ii) absence of parathyroid Klotho alone does not contribute to the development of renal sHPT; iii) in the absence of parathyroid Klotho, the calcineurin-NFAT pathway is constitutively active and mediates the suppression by FGF23 of PTH secretion. The activation of this compensatory mechanism in Klotho-deficient parathyroids might explain the lack of a hyperparathyroid phenotype in *PTH-KL* ^−/−^ mice.

We postulated the existence of a Klotho-independent signaling pathway of FGF23 in the parathyroids based on the findings that *PTH-KL* ^−/−^ mice had unaltered serum PTH levels, a intact parathyroid response to FGF23 injections and that hyperparathyroidism was not aggravated in the setting of renal failure despite an effective ablation of parathyroid Klotho and an apparent lack of MAPK activation in response to FGF23. Because FGF23 was recently shown to promote pathological growth of the myocardium and proposed as a mediator of left ventricular hypertrophy through calcineurin-dependent mechanisms [16], we speculated that this pathway might also be activated by FGF23 in the parathyroids. Indeed, there was a marked upregulation of the calcineurin-modulator MCIP1 and a more prominent nuclear localization of NFATC2 in *PTH-KL* ^−/−^ mice treated with FGF23, strongly supporting a compensatory activation of the calcineurin-NFAT pathway. The functional importance of this pathway was confirmed *in vivo* and *ex vivo* where pretreatment with the calcineurin inhibitor CsA abolished the PTH response to FGF23 treatment in *PTH-KL* ^−/−^ mice but not in wild-type mice. Importantly, our data are also consistent with a previous report demonstrating increased PTH transcript levels in calcineurin Aβ *null* mice and posttranslational regulation of PTH *in vitro* by CsA [21]. The blunted PTH response to FGF23 injection in *PTH-KL^−/−^* mice indicates that parathyroid FGF23 signaling is dependent on two principal pathways, Klotho-FGFR activation and calcineurin activation respectively. However, the exact intra-cellular mechanisms mediating FGF23 suppression of PTH remain to be defined. Importantly, the expression of FGFR1, which has been implicated as the relevant FGFR for parathyroid FGF23 signaling, was unaltered in *PTH-KL^−/−^* mice. This supports that their FGF23-driven calcineurin activation occurs without a compensatory increase in its target FGFR.

The identification of a novel FGF23-calcineurin signaling pathway in the parathyroid glands has several implications. First, it challenges the current concepts of FGF23-mediated PTH regulation and secretion. Second, it raises the possibility of previously neglected Klotho-independent down-stream effects of FGF23. This should be put in the context that abnormally high levels of FGF23 in humans are associated with adverse clinical outcomes including cardiovascular disease, CKD progression rate and mortality [22], [23], [24], [25], [26], [27], [28]. Indeed, FGF23 may contribute to pathological left ventricular hypertrophy in a Klotho-independent manner [16]. Future studies are warranted to explore Klotho-independent FGF23 signaling at the systemic level.

Although FGF23 appears to signal independently of Klotho in both parathyroid glands and cardiomyocytes [16] there is a fundamental difference in its signaling in these tissues. In the heart, FGF23 signaling is an ‘off-target’ effect in a non-classical, non-Klotho expressing organ. In contrast, in parathyroid glands it may rather be viewed as an ‘on-target’ effect in a Klotho-expressing target organ which represents a paradigm shift in the conceptual framework of FGF23 endocrine action.

SHPT is an inevitable complication in CKD patients with advanced renal failure. Serum FGF23 concentrations rise in parallel with a decline in glomerular filtration rate and are markedly elevated at later stages of CKD [29]. Because PTH stimulates FGF23 synthesis in bone and FGF23 signals back to the parathyroids and suppresses PTH secretion [30], a reasonable assumption is that FGF23 rises in CKD in part to counteract sHPT. Indeed, rats subjected to parathyroidectomy prior to induction of renal failure did not respond with increased FGF23 levels [30]. However, the unabated development of sHPT in the face of extreme FGF23 elevations poses a dilemma: either the inhibitory action of FGF23 on PTH is not relevant *in vivo* or the parathyroids may lack responsiveness to FGF23 in CKD. The latter option is supported by studies in CKD rat models demonstrating an attenuated or abolished parathyroid response to FGF23 [9], [10]. Parathyroid FGF23 resistance corroborates data showing a reduction in both Klotho and FGFRs in animal models and in human-derived surgically resected hyperplastic parathyroid glands [11], [12]. Our data unequivocally support that parathyroid FGF23 resistance is not primarily induced by Klotho insufficiency. Speculatively, reduced expression of the cognate FGFR(s) could be the principal mechanism underlying this phenomenon.

**Figure 5:**
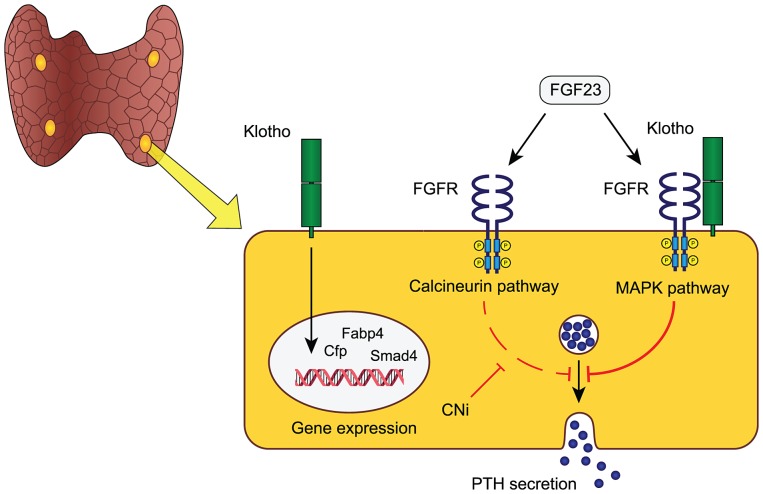
Proposed model of FGF23-Klotho function in parathyroid glands. FGF23 is an endocrine ligand that in the parathyroid glands either binds to an FGF-receptor and membrane-bound Klotho, to elicit activation of the MAPK-pathway, or acts via a calcineurin-dependent signaling pathway. Both pathways induce a rapid inhibition of PTH secretion within 15 minutes. The MAPK-pathway is likely the dominant pathway in physiology, although their relative contributions are unknown. In addition to mediating MAPK-dependent FGF23 signaling, Klotho may play an intrinsic role in modulating parathyroid transcriptional activity for sustained demands and long-term parathyroid function. Speculatively, the clinical use of calcineurin inhibitors (CNi) that block calcineurin signaling may increase the susceptibility to develop, or aggravate pre-existing, hyperparathyroidism in patients with reduced Klotho level such as in chronic kidney disease and primary hyperparathyroidism.

Another potentially relevant implication of our findings is that a large portion of CKD patients receiving a renal allograft suffer from persistent sHPT unproportional to their residual kidney function in the face of high systemic FGF23 [31]. We speculate that this might be due to a dual blocking of FGF23 signaling, both through the endogenously decreased abundance of parathyroid FGFRs/Klotho and the superimposed inhibition of the calcineurin-NFAT pathway by calcineurin inhibitors (e.g. cyclosporine and tacrolimus) commonly used as immunosuppressive agents. This hypothesis and the proposed role of parathyroid resident Klotho is modeled in Figure 5.

The role of parathyroid Klotho remains controversial due to previous conflicting data. Klotho was reported to modulate parathyroid Na^+^/K^+^-ATPase activity via direct interaction with its α1-subunit causing an increased abundance of plasma membrane Na^+^/K^+^-ATPase, which in turn promotes PTH secretion through an increased electrochemical gradient [7]. However, this mechanism was only functional at low extra-cellular calcium concentrations and another study demonstrated that blocking of the Na^+^/K^+^-ATPase with the digitalis glycoside ouabain did not affect the PTH secretory response to acute hypocalcemia [8]. This study underscores that parathyroid Klotho does not alter parathyroid sensitivity to acute fluctuations in serum calcium.

Given the apparent normal phenotype of *PTH-KL^−/−^* mice and their intact response to FGF23 it remains uncertain what other functions parathyroid Klotho may have. The cause of increased serum 1,25(OH)_2_D in *PTH-KL^−/−^* mice is unknown since expression levels of *Cyp27b1* and *Cyp24a1* are unaltered in kidneys and parathyroid glands. Yet, it is conceivable that parathyroid Klotho has a functional impact on vitamin D metabolism in *PTH-KL^−/−^* mice and that their elevated 1,25(OH)_2_D level contributes to normalizing PTH and counteracts development of hyperparathyroidism. Further, we speculate that Klotho is an important local transcriptional regulator of genes involved in sustained challenge of parathyroid function or having a long-term impact on parathyroid function. In support of this idea, *PTH-KL* ^−/−^ mice had altered transcript levels of several transcriptional regulators including *Cfd*, *Smad4*, *Fabp4* and *Pin1*. The increased level of *Pin1* in *PTH-KL^−/−^* mice is intriguing given its role in destabilizing PTH mRNA [32] and may represent yet another protective intracellular mechanism against hyperparathyroidism when FGF23-Klotho signaling is chronically disrupted.

In sum, we dissected the physiological and pathophysiological role of parathyroid Klotho and elucidated several critical and novel aspects in this regard. Most importantly, our study uncovered a Klotho-independent FGF23 signaling mechanism in parathyroid glands with potential implications for multiple disorders of mineral metabolism, especially in CKD.

## Materials and Methods

### Generation of parathyroid-specific Klotho knockout mice

Mice with a parathyroid-specific Klotho deletion were generated using Cre-Lox recombination as previously described [33]. Briefly, loxP sequences were introduced in the flanking regions of exon 2 of the Klotho gene. Floxed mice were crossed with mice expressing Cre recombinase under the human PTH promotor (129;FVB-Tg(PTH-cre)4167Slib/J; Jackson laboratory, US) and the offspring subsequently analyzed. Floxed littermates not expressing Cre were used as wild-type controls.

### Genotyping

Total DNA was extracted from tail biopsies and genotyping performed with standard PCR techniques.

### Animal housing

Mice were fed a standard chow (RM1, SDS, UK) containing 0.73% calcium and 0.52% phosphorous. All animals had free access to food and drinking water. Blood sampling was performed by tail vein incision at intermediate time points and through the axillary artery at sacrifice. Animals were fasted 4 hour prior to blood sampling. All experiments were conducted in compliance with the guidelines of animal experiments at Karolinska Institutet and approved by the regional ethical board (Stockholm South ethical committee).

### Serum and urine biochemistries

Calcium, phosphorous and creatinine were measured using quantitative colorimetric assay kits (BioChain, US). Urine values were multiplied by 1000. Serum PTH was measured using a Mouse Intact PTH ELISA kit (Immutopics, US). For the FGF23 injection experiments the newer Mouse 1–84 PTH ELISA kit (Immutopics) and plasma samples were used. Serum 1,25-dihydroxy vitamin D was measured using a RIA kit (IDS, US). Intact FGF23 was measured using an FGF23 ELISA kit (Kainos Laboratories, Japan).

### RNA isolation and transcript analysis

Parathyroid glands and surrounding adhesive thyroid tissue were carefully removed in conjunction with sacrifice and immediately frozen in OCT. The parathyroid tissue was then dissected using laser capture microdissection. Thirty µm thick cryo-sections were cut and mounted on PEN foil–coated slides (Leica, Germany). Sections were stained with the Arcturus histogene kit (Applied Biosystems, US) and microdissected with a Leica ASLMD microscope (Leica). Total RNA was extracted by the Arcturus picopure RNA isolation kit (Applied Biosystems). The nanostring nCounter system (Nanostring technologies, US) was used to obtain mRNA expression profiles. Data were processed using several normalization strategies, including quantile normalization and 6 house-keeping genes.

### Immunohistochemistry and immunofluorescence

Thyro-parathyroid tissue were dissected, fixed in 4% paraformaldehyde and embedded in paraffin. 4 µm sections were immersed in 3% H_2_O_2_ in methanol, treated with 4% normal serum and blocked with Avidin and Biotin (Vector Laboratories, US). Sections were incubated with primary antibodies at 4°C overnight. For immunohistochemistry slides were incubated with biotinylated secondary antibodies followed by Vector ABC Reagent and developed with DAB substrate (Vector Laboratories). For immunofluorescence, Alexa Fluor conjugated secondary antibodies were used for visualization (Invitrogen, US). The primary antibodies used were rat monoclonal anti-Klotho (KM2076, TransGenic Inc. Japan) mouse monoclonal anti-CaSR (NB120-19347, Novus Biologicals, US), mouse monoclonal anti-VDR (sc-13133, Santa Cruz Biotechnology, US), rabbit monoclonal anti-Ki67 (SP6, Thermo Scientific, US), rabbit monoclonal anti-Erk1/2 and anti-phospho-Erk1/2 (Cell Signaling, US), mouse monoclonal anti-NFATc2 (sc-7295, Santa Cruz Biotechnology) and rabbit polyclonal anti-MCIP1 (a kind gift from Dr. Christian Faul) [34].

### Bone phenotyping

Processing of undecalcified bone specimens and cancellous bone histology in the distal femoral metaphysis were performed according to standard protocols. The area within 0.25 mm from the growth plate was excluded from the measurements. µCT were performed using a Scanco Medical µCT 35 system (Scanco, Switzerland).

### Determination of parathyroid calcium sensitivity

Investigation of the PTH-calcium relationship was performed by intraperitoneal injections with calcium-gluconate (300 µmol/kg) or EGTA (450 µmol/kg) dissolved in sterile saline as described elsewhere [14]. Blood samples were collected from tail vein for serum PTH and calcium measurements after 30 minutes.

### Induction of renal failure

We employed an adenine-based model of renal failure in which mice were given adenine mixed in a casein containing diet. Mice were exposed to 0.3% adenine during the first 7 days followed by 0.2% adenine for 11 days, and finally 0.1% adenine for the rest of the study. Adenine was purchased from Sigma-Aldrich (US), and the powdered casein-based diet from Special Diets Services.

### Intravenous injections of FGF23 and pretreatment with CsA

Recombinant human FGF23 protein (A28 to I251) was produced as previously described [35], [36]. In all experiments, FGF23 was injected intravenously through the tail vein after a 4-hour fasting period at the dose 0.15 mg/kg dissolved in 300 µl saline. Blood samples were drawn 15 min after injection of FGF23, and the animals were then sacrificed and thyro-parathyroid tissue immediately excised and fixed in 4% formalin for further analysis. Mice were gavage fed with CsA at the dose 60–150 mg/kg (Sigma-Aldrich) two hours prior to FGF23 injection.

### Organ culture

During anesthesia the trachea was severed superiorly and inferiorly to the thyroid gland. The trachea-thyro-parathyroid complex was excised and placed in 1 ml serum-free medium (DMEM/F-12, HEPES supplemented with insulin, transferrin and BSA) as previously described [20]. Medium was replaced daily for 4 days before the experiment started, in accordance with the protocol by Ritter et al. On day 4 fresh medium was added and the tissue incubated for 2 h ( = baseline). The medium was then replaced with regular medium ( = control) or medium containing 10 ng/mL FGF23 and incubated for 2 h. One group was pre-treated with medium containing CsA (0.83 µM) for 2 h prior to incubation with FGF23. All values were calculated as relative change compared to baseline.

### Statistical analysis

GraphPad Prism 5.0 (GraphPad Software Inc, US) was used for statistical analysis. Variables were tested with either two-tailed t-test or Mann-Whitney test. Correlations between serum PTH and calcium levels were tested with linear regression analysis.

## Supporting Information

Figure S1 Tissue-specific deletion of the *Klotho* gene. *Left panel*. LoxP sites were inserted into intron 1 and 2 of Klotho, enabling targeted disruption of the gene function. Klotho deletion was restricted to parathyroid glands by using transgenic mice expressing Cre recombinase driven by the *PTH* gene promoter. *Right panel*. Representative genotyping by PCR to confirm presence of floxed Klotho alleles.
(PDF)

Figure S2 Bone phenotype of 6 week-old wild-type and *PTH-KL^−/−^* mice. There were no significant changes in bone mineral density or skeletal histology in *PTH-KL^−/−^* mice compared to wild-type mice. BV/TV: Distal Femoral Bone Volume/Total Volume. Tb.N: Trabecular Number. Tb.Th: Trabecular Thickness. Tb.Sp: Trabecular Spacing. M.BV/TV: Midshaft Bone Volume/Total Volume. C.Th: Cortical Thickness.
(PDF)

Figure S3 Representative immunohistochemical/immunofluorescence staining of thyro-parathyroid tissue from *PTH-KL^−/−^* mice and wild-type controls. A) Proliferation rate as determined by Ki67 index was unaltered in *PTH-KL^−/−^* mice (40× magnification). B) Dual immunofluorescence staining showed that Klotho co-localized with PTH, CaSR and VDR although the expression level of these proteins appeared quantitatively unaltered in Klotho-deleted cells versus adjacent Klotho-expressing cells (40× magnification).
(PDF)

Figure S4 Calcium – PTH relationship. Mice were injected intraperitoneally with calcium-gluconate or EGTA which causes rapid increments or decrements, respectively, in serum calcium level. Blood samples were drawn after 30 minutes for analysis of serum calcium and PTH. The calcium-PTH relationship in *PTH-KL^−/−^* mice (n = 8) and wild-type mice (n = 5) is presented and did not differ between the genotypes.
(PDF)

Table S1 Renal gene expression in *PTH-KL^−/−^* mice and their wild-type littermates. All transcripts were normalized to *Beta-actin*. N = 8 for each genotype.
(PDF)

Table S2 A nanostring array encompassing >90 genes critical for parathyroid function. N = 3 for each genotype. Significant results (p<0.05) are in bold.
(PDF)

Table S3 Serum and urine biochemistries in *PTH-KL^−/−^* and wild-type mice after induction of renal failure.
(PDF)
