# Supplementary material for: Parathyroid-Specific Deletion of Klotho Unravels a Novel Calcineurin-Dependent FGF23 Signaling Pathway That Regulates PTH Secretion
Source: PLoS Genet. 2013 Dec 12;9(12):e1003975. doi: 10.1371/journal.pgen.1003975 (PMC3861040; doi:10.1371/journal.pgen.1003975)
Supplement: Figure S1 — Tissue-specific deletion of the Klotho gene. Left panel. LoxP sites were inserted into intron 1 and 2 of Klotho, enabling targeted disruption of the gene function. Klotho deletion was restricted to parathyroid glands by using transgenic mice expressing Cre recombinase driven by the PTH gene promoter. Right panel. Representative genotyping by PCR to confirm presence of floxed Klotho alleles. (PDF) [file pgen.1003975.s001.pdf]

Figure S1.

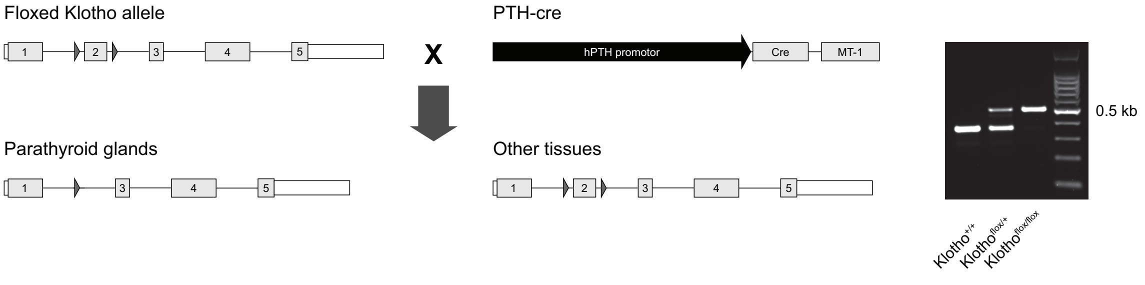
Floxed Klotho allele
1
2
3
4
5
X
PTH-cre
hPTH promotor
Cre
MT-1
Parathyroid glands
1
3
4
5
Other tissues
1
2
3
4
5
0.5 kb
Klotho+/+
Klothoflox/+
Klothoflox/flox
