# Supplementary figures and images for: Parathyroid-Specific Deletion of Klotho Unravels a Novel Calcineurin-Dependent FGF23 Signaling Pathway That Regulates PTH Secretion
Source: PLoS Genet. 2013 Dec 12;9(12):e1003975. doi: 10.1371/journal.pgen.1003975 (PMC3861040; doi:10.1371/journal.pgen.1003975)

**Figure S2.**

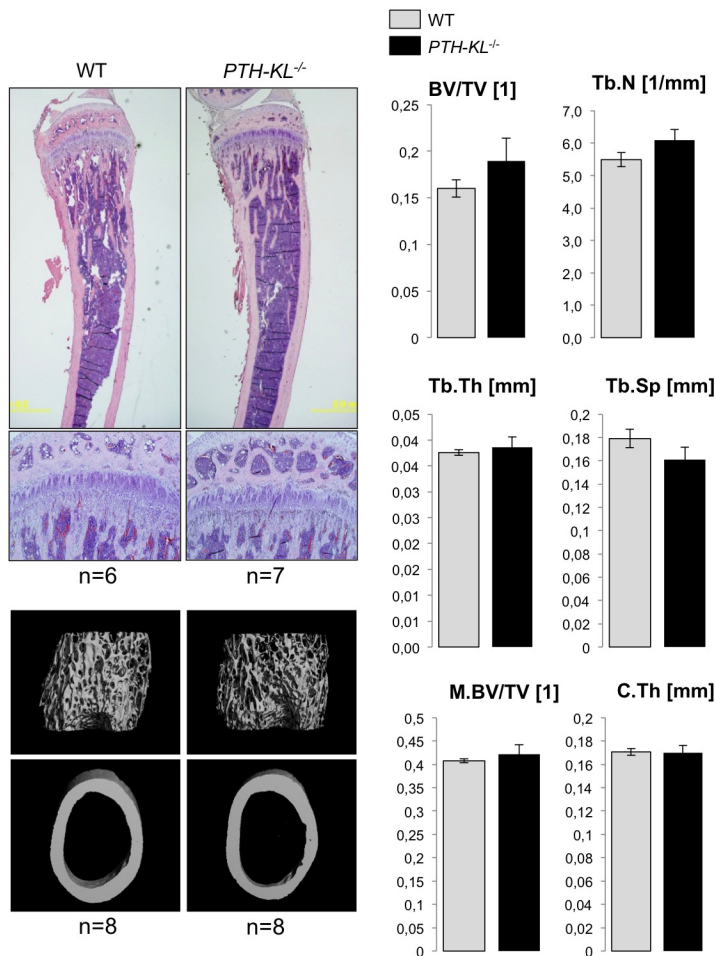

Supplement: Figure S2 — Bone phenotype of 6 week-old wild-type and PTH-KL−/− mice. There were no significant changes in bone mineral density or skeletal histology in PTH-KL−/− mice compared to wild-type mice. BV/TV: Distal Femoral Bone Volume/Total Volume. Tb.N: Trabecular Number. Tb.Th: Trabecular Thickness. Tb.Sp: Trabecular Spacing. M.BV/TV: Midshaft Bone Volume/Total Volume. C.Th: Cortical Thickness. (PDF) [file pgen.1003975.s002.pdf]

Figure S3.

A)

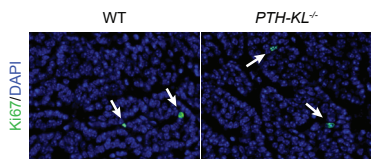

B)

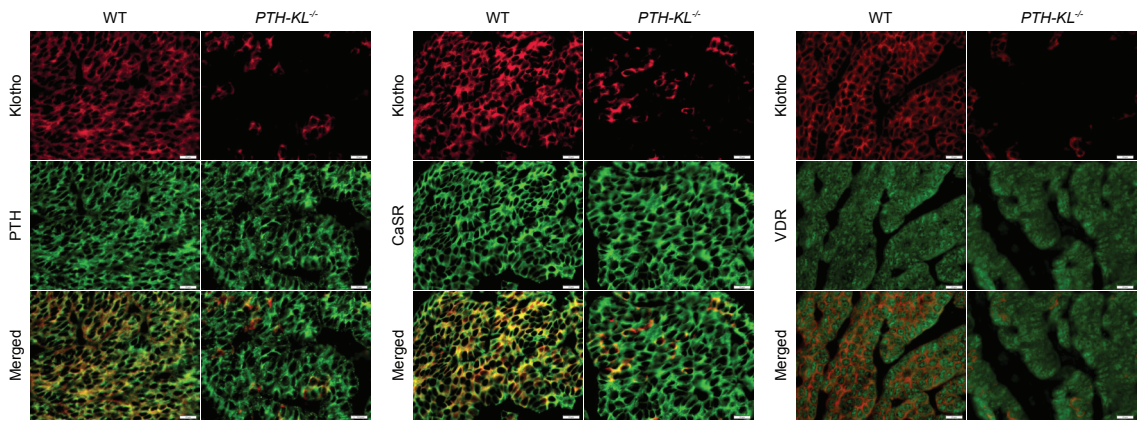

Supplement: Figure S3 — Representative immunohistochemical/immunofluorescence staining of thyro-parathyroid tissue from PTH-KL−/− mice and wild-type controls. A) Proliferation rate as determined by Ki67 index was unaltered in PTH-KL−/− mice (40× magnification). B) Dual immunofluorescence staining showed that Klotho co-localized with PTH, CaSR and VDR although the expression level of these proteins appeared quantitatively unaltered in Klotho-deleted cells versus adjacent Klotho-expressing cells (40× magnification). (PDF) [file pgen.1003975.s003.pdf]

**Figure S4.**

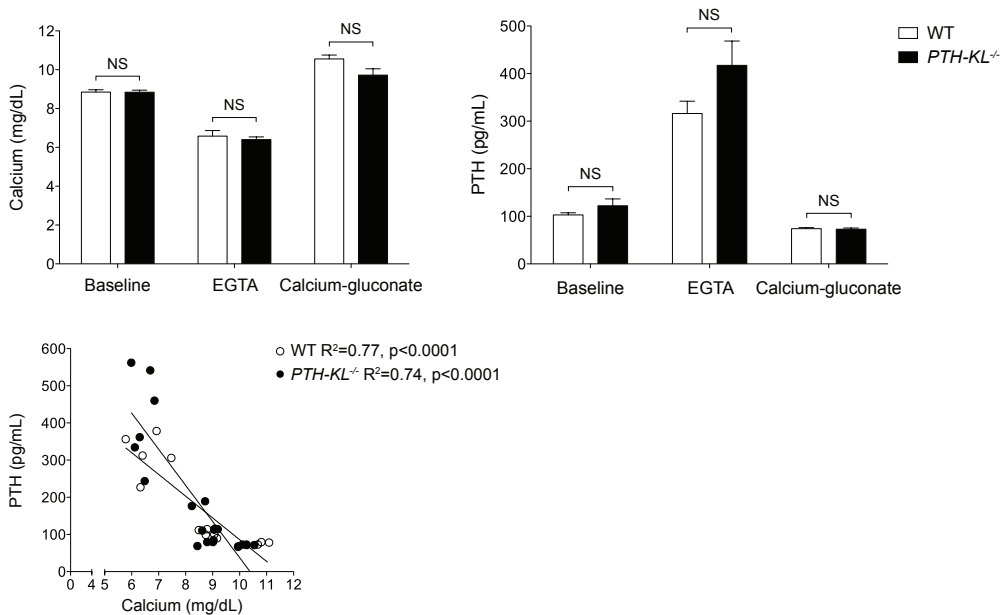

Supplement: Figure S4 — Calcium – PTH relationship. Mice were injected intraperitoneally with calcium-gluconate or EGTA which causes rapid increments or decrements, respectively, in serum calcium level. Blood samples were drawn after 30 minutes for analysis of serum calcium and PTH. The calcium-PTH relationship in PTH-KL−/− mice (n = 8) and wild-type mice (n = 5) is presented and did not differ between the genotypes. (PDF) [file pgen.1003975.s004.pdf]
