# Supplementary material for: Parathyroid-Specific Deletion of Klotho Unravels a Novel Calcineurin-Dependent FGF23 Signaling Pathway That Regulates PTH Secretion
Source: PLoS Genet. 2013 Dec 12;9(12):e1003975. doi: 10.1371/journal.pgen.1003975 (PMC3861040; doi:10.1371/journal.pgen.1003975)
Supplement: Table S1 — Renal gene expression in PTH-KL−/− mice and their wild-type littermates. All transcripts were normalized to Beta-actin. N = 8 for each genotype. (PDF) [file pgen.1003975.s005.pdf]

Table S1.

Gene	Wild-type	*PTH-KL*$^{-/-}$	P-value
Klotho	0.91	0.88	0.74
Npt2a	0.11	0.10	0.66
Cyp27b1	0.89	0.78	0.53
Cyp24a1	0.70	1.64	0.27
TRPV5	0.38	0.39	0.84
VDR	1.35	1.39	0.85
CaSR	1.01	0.92	0.56
