# Supplementary material for: Parathyroid-Specific Deletion of Klotho Unravels a Novel Calcineurin-Dependent FGF23 Signaling Pathway That Regulates PTH Secretion
Source: PLoS Genet. 2013 Dec 12;9(12):e1003975. doi: 10.1371/journal.pgen.1003975 (PMC3861040; doi:10.1371/journal.pgen.1003975)
Supplement: Table S2 — A nanostring array encompassing >90 genes critical for parathyroid function. N = 3 for each genotype. Significant results (p<0.05) are in bold. (PDF) [file pgen.1003975.s006.pdf]

Table S2.

**Gene**	**Wild-type**	***PTH-KL$^{-/-}$***	**P-value**
ADAM 10	100.4	109.2	0.84
ADAM 17	48.3	72.3	0.54
ANK	150.5	116.6	0.27
ATF4	154.5	189.6	0.58
Atp1a1	256.2	263.3	0.93
ATP1b1	114.1	124.5	0.90
ATP1b2	78.9	39.3	0.40
ATP1b3	209.3	240.5	0.46
Bcl2	61.7	89.3	0.11
BMP3	42.5	29.0	0.69
BMP7	75.4	42.0	0.25
C-Myc	52.9	72.3	0.38
Calbindin D9	139.2	139.2	1.00
Caspase3	91.1	99.7	0.63
Caspase9	40.6	39.1	0.93
CaSR	4310.9	3660.1	0.70
Cbfb	303.1	280.4	0.58
Cebpa	74.1	95.0	0.40
CEP63	58.1	68.8	0.60
**Cfd**	**31.7**	**241.8**	**0.00**
Col1a1	243.7	245.6	0.99
CREBbp	94.9	149.2	0.13
CyclinD1	37.4	42.7	0.79
Cyp24a1	17.7	11.7	0.71
Cyp27b1	31.3	23.4	0.72
Dll1 delta-like 1	32.5	25.2	0.65
Ebf1	47.6	11.4	0.18
Egr1	71.7	31.0	0.19

Esr1	65.7	36.5	0.44
Ezrin	391.7	356.6	0.38
**Fabp4**	**40.2**	**206.6**	**0.01**
Fgf13	55.8	80.8	0.61
Fgf17	35.9	37.8	0.95
Fgf20	77.4	41.5	0.08
Fgf6	45.8	38.4	0.84
FGFR1	55.2	63.1	0.85
FGFR2c	150.0	78.8	0.29
Fgfrl1	36.6	35.9	0.98
Gata3	532.1	434.5	0.49
GCM2	327.7	225.5	0.49
Ggcx	85.7	80.6	0.80
HAND1	41.9	12.7	0.15
HDAC1	209.2	201.3	0.87
HDAC2	169.6	223.8	0.16
HDAC3	98.8	74.1	0.38
HDAC4	58.3	48.8	0.68
Hes1	38.0	20.1	0.21
HHIP	189.0	318.3	0.43
HIF	486.7	532.3	0.48
HMGA2	63.1	42.6	0.64
Id1	44.7	82.7	0.13
Id2	77.6	148.1	0.45
IGF1 receptor	65.3	66.2	0.97
IGF2 receptor	43.3	50.7	0.75
**Klotho alpha**	**1850.9**	**564.4**	**0.01**
Mapk1 Mapk2 Erk2 p42	519.4	525.1	0.94
Mapk3 p44 Erk1	135.7	117.2	0.53
MEF2a	61.7	70.7	0.72
MGP	503.4	556.2	0.81

MMP2	67.3	62.3	0.82
Msx2	39.6	23.8	0.44
NCX1	64.8	47.3	0.67
NHERF1 Slc9a3r1	137.4	122.4	0.49
NPT4 slc17a3	33.3	27.2	0.84
Osteopontin	9788.1	5110.6	0.32
P27	334.3	341.8	0.97
PAX1	334.0	395.9	0.71
PAX9	211.4	152.4	0.43
Pit2 slc20a2	55.8	33.1	0.20
PMCA 1b	149.0	111.2	0.11
PRLR	94.6	147.0	0.33
Ptch1	83.9	57.6	0.14
Ptch2	34.0	26.3	0.74
PTH	1017561.9	706914.9	0.54
PTHR1	33.4	19.5	0.67
RANK	30.5	39.2	0.52
Rb	128.4	124.1	0.90
RXR beta	82.2	128.7	0.19
SCMH1	43.4	71.8	0.08
SFRP-1	41.1	58.5	0.58
SFRP-5	142.4	89.2	0.48
Sfrp4	66.6	52.5	0.58
Smad1	71.7	57.5	0.39
Smad2	53.7	65.2	0.52
**Smad4**	**202.0**	**163.6**	**0.03**
Smad6	121.1	66.8	0.25
Smo	60.2	69.6	0.36
Sox3	32.9	23.3	0.73
Sox9	110.2	129.4	0.59
Tbce	66.9	54.3	0.47

TGF beta	68.4	73.2	0.81
VDR	507.0	321.0	0.24
Wnt3a	37.8	21.9	0.65
Wnt5a	185.2	127.3	0.44
Wnt5b	48.5	48.4	1.00
Zfp219	35.2	30.4	0.63
Zfp423	42.5	37.5	0.81

**Reference genes**	**Wild-type**	***PTH-KL***$^{-/-}$	**P-value**
Beta-actin	3063.7	4862.2	0.51
GAPDH	423.9	471.5	0.58
Gusb	138.5	124.5	0.62
Hmbs	114.4	75.5	0.20
Hprt1	91.1	111.6	0.51
