# Supplementary material for: Parathyroid-Specific Deletion of Klotho Unravels a Novel Calcineurin-Dependent FGF23 Signaling Pathway That Regulates PTH Secretion
Source: PLoS Genet. 2013 Dec 12;9(12):e1003975. doi: 10.1371/journal.pgen.1003975 (PMC3861040; doi:10.1371/journal.pgen.1003975)
Supplement: Table S3 — Serum and urine biochemistries in PTH-KL−/− and wild-type mice after induction of renal failure. (PDF) [file pgen.1003975.s007.pdf]

Table S3.

**Serum biochemistries**	**Wild-type (n=6)**	***PTH-KL*$^{-/-}$ (n=8)**
Calcium (mg/dL)	9.28 (±1.16)	9.40 (±1.04)
Phosphorous (mg/dL)	11.86 (±2.23)	12.91 (±1.52)
Creatinine (mg/dL)	0.78 (±0.06)	0.75 (±0.06)
FGF23 (pg/mL)	2627 (706-13 610)	3090 (610-15 732)
**Urine biochemistries**		
Calcium/creatinine	0.97 (±0.23)	1.17 (±0.27)
Phosphate/creatinine	52.2 (±11.5)	49.9 (±8.1)

Urine values are multiplied by 1000. Data is presented as mean (± SEM). There were no significant differences between the groups.
